# A scoping review of the effects of mushroom and fungus extracts in rodent models of depression and tests of antidepressant activity

**DOI:** 10.3389/fphar.2024.1387158

**Published:** 2024-06-03

**Authors:** Catherine K. Wang, Gio Kim, Lily R. Aleksandrova, William J. Panenka, Alasdair M. Barr

**Affiliations:** ^1^ Department of Anesthesiology, Pharmacology and Therapeutics, Faculty of Medicine, University of British Columbia (UBC), Vancouver, BC, Canada; ^2^ British Columbia Mental Health and Substance Use Services Research Institute, Vancouver, BC, Canada; ^3^ Department of Psychiatry, Faculty of Medicine, Canada Faculty of Pharmaceutical Sciences, UBC, Vancouver, BC, Canada

**Keywords:** animal model, antidepressant, fungus, mushroom, preclinical

## Abstract

One of the most important developments in psychopharmacology in the past decade has been the emergence of novel treatments for mood disorders, such as psilocybin for treatment-resistant depression. Psilocybin is most commonly found in different species of mushroom; however, the literature on mushroom and fungus extracts with potential antidepressant activity extends well beyond just psilocybin-containing mushrooms, and includes both psychedelic and non-psychedelic species. In the current review, we systematically review the preclinical literature on mushroom and fungus extracts, and their effects of animal models of depression and tests of antidepressant activity. The PICO structure, PRISMA checklist and the Cochrane Handbook for systematic reviews of intervention were used to guide the search strategy. A scoping search was conducted in electronic databases PubMed, CINAHL, Embase and Web of Science. The literature search identified 50 relevant and suitable published studies. These included 19 different species of mushrooms, as well as seven different species of other fungi. Nearly all studies reported antidepressant-like effects of treatment with extracts. Treatments were most commonly delivered orally, in both acute and chronically administered studies to predominantly male rodents. Multiple animal models of depression were used, the most common being unpredictable chronic mild stress, while the tail suspension test and forced swim test were most frequently used as standalone antidepressant screens. Details on each experiment with mushroom and fungus species are discussed in detail, while an evaluation is provided of the strengths and weaknesses of these studies.

## Introduction

Mood disorders remain among the most prevalent and disabling of all psychiatric conditions. They also represent one of the leading causes of worldwide disease burden (Friedrich, 2017; Collaborators, 2022). While many individuals affected by mood disorders respond well to treatment, a significant proportion of people show either partial or no response to antidepressant therapies (McLachlan, 2018). If an individual fails to respond to two or more trials of standard antidepressant pharmacotherapy, they may be considered “treatment-resistant” (Voineskos et al., 2020). Furthermore, many individuals may show a therapeutic response to antidepressant treatment but suffer side-effects that significantly reduce their quality of life (Teng et al., 2022), resulting in reduced treatment adherence (Hung, 2014; Rossom et al., 2016).

Clinical treatment options for those who do not respond well to standard antidepressant therapies have historically remained limited. However, in recent years, several landmark studies have reported that administration of psychedelic drugs under controlled conditions, typically in combination with psychotherapy, can significantly reduce depressive symptoms (Griffiths et al., 2016; Ross et al., 2016; Palhano-Fontes et al., 2019; Davis et al., 2021; Eisenstein, 2022; Goodwin et al., 2022). Importantly, this includes individuals with treatment-resistant depression (Carhart-Harris et al., 2016). Additionally, in clinical trials reported to-date, the side-effect profile of these compounds has appeared relatively benign (Eisenstein, 2022) with no evidence of some of the side-effects associated with other psychotropic medications, such as weight gain and metabolic dysregulation (Boyda et al., 2013; Boyda et al., 2021; Sepúlveda-Lizcano et al., 2023).

While the use of the term “psychedelic” has no official definition, it typically refers to a drug that is able to alter perception, thoughts, feelings and consciousness in humans (Hosanagar et al., 2021). Psychedelic drugs are commonly categorized as either “classical” or “atypical” (Kamal et al., 2023). The former category represents drugs with agonism or partial agonism at the serotonergic 5-HT2A receptor, and includes tryptamines (such as psilocybin and DMT), ergolines (such as LSD) and phenethylamines (such as mescaline) (Kelmendi et al., 2022). The atypical psychedelics have diverse mechanisms of action (Aleksandrova and Phillips, 2021), which are not primarily at the 5-HT2A receptor, and include drugs such as ketamine, ibogaine, muscimol and salvinorin A (Kelmendi et al., 2022). At this point, it is important to note that many compounds from both classes of psychedelic drugs have their origins in commonly available mushrooms and other fungi.

### Mushrooms and other fungi

Mushrooms are generally defined as the spore-producing fruiting body of a fungus. Traditional medicine has used mushrooms, and fungi in general, in medical treatment for centuries (Yadav and Negi, 2021; Gravina et al., 2023), taking advantage of their numerous perceived therapeutic benefits. Such properties have been reported to include antimicrobial (Moussa et al., 2022), antibacterial, antioxidant, hepatoprotective (Venturella et al., 2021), and antitumor (Pandya et al., 2019) effects. More recently, researchers have investigated “medicinal” mushrooms as potential alternatives or complements to mainstream antidepressant treatments. For example, non-psychedelic species such as *Hericium erinaceus* and *Ganoderma lucidum* have been noted as having mood-improving qualities in humans (Nagano et al., 2010; Fijałkowska et al., 2022), although head-to-head trials comparing effects against standard antidepressant pharmacotherapies are lacking. Nevertheless, the increasing body of evidence which indicates that psilocybin (a psychedelic compound found in many species of mushrooms (Strauss et al., 2022)) has potent antidepressant effects, including in those with treatment-resistant depression (Haikazian et al., 2023; Simonsson et al., 2023), supports the notion that mushrooms and other fungi may hold significant therapeutic potential in this area. However, given the enormous number of potential species of mushroom and other fungi that could have antidepressant effects, measured against the tremendous costs associated with conducting clinical trials in humans, it is critical to determine which mushroom and fungus species and their derivatives represent the best preclinical leads for further development. In this context, it is vitally important to understand which species have already demonstrated efficacy in preclinical animal models of depression and specific screens for antidepressant activity. The purpose of the present scoping review is therefore to systematically identify which mushroom and fungus species have been tested for antidepressant effects in specific preclinical models, and to summarize and evaluate the results of these studies.

## Materials and methods

The PICO structure, PRISMA checklist and the Cochrane Handbook (Higgins and Green, 2011) for systematic reviews of intervention were used to guide the search strategy. A scoping search was conducted in electronic databases PubMed, CINAHL (via EBSCO), Embase (via Ovid), and Web of Science, as previously (Tse et al., 2014; Yuen et al., 2021; Lian et al., 2022). One preprint source was found as a suggestion under another article and later located on Google Scholar. The latest literature search was conducted on 19 December 2023.

A combination of 26 individual search terms were used with the following keywords: “mushroom” or “mushrooms” or “fungus” and “depress*” or “antidepress*” and “animal” or “animal model”. Filters excluding human studies or non-article sources were applied as needed. Searches were also conducted using specific behavioural models/tests or mushroom species as keywords. Studies were limited to those using rodent species as those reflect the expertise of the authors (Lu et al., 2005; Barr et al., 2006; Hill et al., 2007; Boyda et al., 2014); however, it is important to note that other species, such as zebrafish, represent additional valid animal models of antidepressant efficacy (Braun et al., 2024).

Studies were included if they met the following criteria: 1) studies tested a mushroom, fungus, or relevant mushroom derivative, and; 2) used a rodent model or behavioural test of depression or screen of antidepressant activity. Studies were excluded if they 1) were not published in English, or; 2) were not full text original research studies (i.e., conference abstracts, review papers).

**FIGURE 1:**
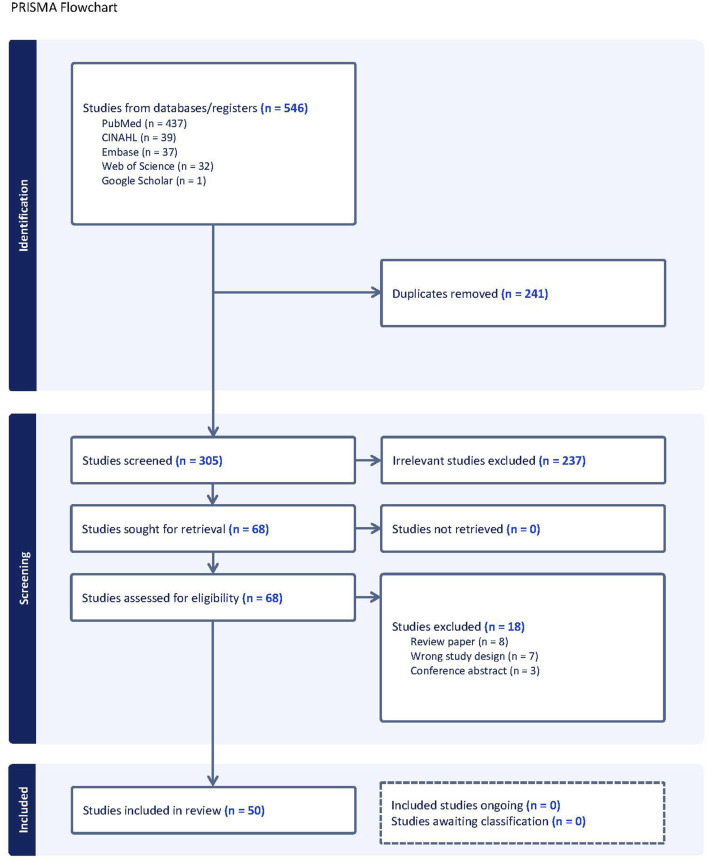
Literature review flow process.

A total of 546 articles were identified using Covidence (www.covidence.org), with 241 duplicates removed, leaving 305 articles to be screened. After title and abstract screening, 237 were deemed irrelevant, leaving 68 studies for eligibility assessment. After full text review, 18 studies were excluded, leaving 50 studies in the final database. Figure 1 outlines a PRISMA flowchart of the study selection process.

Abstracts and full texts were screened by GK and CKW. Data was extracted independently by GK and CKW with key variables extrapolated and outlined in Supplementary Table S1. Any discrepancies throughout the process were brought to consensus by GK and CKW with the assistance of AMB if required.

## Results

The literature search identified 50 relevant and suitable published studies. These included 19 different species of mushrooms see Table 1, as well as seven different species of other fungi see Table 2; there were also three studies that used compounds which are common to multiple mushroom species.

**TABLE 1:** Summary of rodent depression models and behavioural tests used to screen for antidepressant effects in different mushroom species. Subchronic and chronic treatment schedules include daily administration of drug unless otherwise stated.

Route	Treatment duration	Extraction method	Rodent species	Strain	Sex	Sample size	Behavioural test or model	Test duration	Test frequency	Doses tested	Combined with	Other notes	Reference number
*Hericium erinaceus*													
Food	Chronic (92 d)	Methanol	Rat	Wistar	Female	Sham (n = 11) OVX Model (n = 11) OVX + HE (n = 10) OVX + E_2_ (n = 8)	OVX	96 d	Once	n/a	n/a	Models menopausal depression	Anuar et al. (2022)
							FST	5 min		OVX Model* 1%^#^			
p.o.	Chronic (28 d)	Ethanol	Mouse	ICR	Male	n = 10 per group Control CRS Model CRS + HE (Low, Medium, High)	CRS	14 d	2 h daily	n/a	Erinacine A	Mycelium	Chiu et al. (2018)
							FST	5 min	Once	CRS Model*** 100 mg/kg 200 mg/kg^##^ 400 mg/kg^##^			
							TST						
i.p.	Chronic (28 d)	Ethanol	Mouse	C57BL/6	Male	n = 76 in total	CRS	14 d	6 h daily	n/a^#^	n/a	n/a	Chong et al. (2021)
							SPT	2 h	Once	CRS Model*** 10 mg/kg^##^ 25 mg/kg^##^			
							TST	5 min		CRS Model* 10 mg/kg 25 mg/kg^##^			
s.c.	Chronic (21 d)	Alcohol	Mouse	SAMP8, BALB/C	Male	n = 8 per group CORT Model CORT + (Low, Medium, High) CORT + Fluoxetine	CORT	21 d	Daily	40 mg/kg	Chlorella	0.1 mL chlorella + 6 mg HE 0.2 mL chlorella + 12 mg HE 0.4 mL chlorella + 24 mg HE	Chou et al. (2022)
p.o.							FST	Last 4 min of 6 min	Once	CORT Model 0.25 mL/25 g 0.5 mL/25 g^#^ 2.5 mL/25 g^#^			
p.o.	Chronic (9 d)	n/a	Mouse	C57BL/6	Male	n = 6 Control Low dose High dose	TST	15 min	Daily for 9 d	75 mg/kg 150 mg/kg % immobility increased daily over 9 d period	n/a	Mycelium; Uses TST-induced depression model, not screen	Li et al. (2021b)
p.o.	Chronic (28 d)	Ethanol	Mouse	C57BL/6	Male	n/a Control HE (Low, High)	FST	Last 4 min of 6 min	Once	20 mg/kg* 60 mg/kg*	n/a	n/a	Ryu et al. (2018)
							TST						
i.p.	Subchronic (1 d)	n/a	Mouse	C57BL/6N	Male	n = 11–12 per group Control Control + Amycenone LPS Model LPS + Amycenone	LPS	1 d	Once	n/a	n/a	Amycenone: hericenones/hericium isolates (0.5%) and amyloban (6%) Use LPS to induce depression	Yao et al. (2015)
p.o.							FST	6 min		Non-LPS 200 mg/kg LPS LPS Model** 200 mg/kg^#^			
							TST	10 min		Non-LPS 200 mg/kg LPS LPS Model*** 200 mg/kg^##^			
*Ganoderma lucidum*													
p.o.	Chronic (28 d)	Ethanol	Rat	Sprague-Dawley	Male	n = 8 per group Control UCMS Model UCMS + Gl-E (Low, Medium, High) UCMS + Fluoxetine	UCMS	28 d	Daily	n/a	n/a	Preprint; not peer-reviewed	Cheng (2023)
							SPT	3 h	Once	UCMS Model*** 0.02 g/kg 0.1 g/kg^#^ 0.5 g/kg^##^			
p.o.	Chronic (28 d)	Ethanol	Mouse	Swiss Albino	Both	n = 5 per group Control EEGL (Low, Medium, High)	FST	Last 4 min of 6 min	Once	Male 100 mg/kg* 200 mg/kg** 400 mg/kg** Female 100 mg/kg* 200 mg/kg* 400 mg/kg**	n/a	n/a	Ezurike et al. (2023)
	Chronic (29 d)						TST			Male 100 mg/kg* 200 mg/kg** 400 mg/kg** Female 100 mg/kg* 200 mg/kg* 400 mg/kg*			
i.p.	n/a	n/a	Mouse	C57BL/6	Male	SPT: n = 7 per group TST: n = 8–10 per group FST: n = 9–10 per group Control Control + GLP (Low, Medium, High) CSDS Model CSDS + GLP (Medium) Imipramine	CSDS	10 d	5–10 min daily	n/a	n/a	Polysaccharide	Li et al. (2021a)
							SPT	2 h	Once	CSDS Model***			
	Subchronic (5 d)						FST	Last 4 min of 6 min		Non-CSDS 1 mg/kg 5 mg/kg*** 12.5 mg/kg CSDS 5 mg/kg^###^			
	Acute (60 min)						TST			Non-CSDS 1 mg/kg 5 mg/kg* 12.5 mg/kg CSDS 5 mg/kg^#^			
p.o.	Acute (60 min)	Water	Rat	Sprague-Dawley	Male	Control (n = 8) MAK (Low, High) (n = 6) Imipramine (n = 5)	FST	5 min	Once	0.3 g/kg 1 g/kg*	n/a	Mycelium	Matsuzaki et al. (2013)
i.p.	Chronic (21 d)	Ethanol + Ethyl Acetate	Mouse	C57BL/6J	Male	n = 11–13 per group Control Control + GLT MS Model MS + GLT	MS	21 d	4 h daily	n/a	n/a	Triterpenoids	Mi et al. (2022)
							SPT	24 h	Once	Non-MS 40 mg/kg MS MS Model**** 40 mg/kg^####^			
							FST	6 min		Non-MS 40 mg/kg MS MS Model**** 40 mg/kg^###^			
							TST			Non-MS 40 mg/kg MS MS Model* 40 mg/kg^#^			
							Splash Test	5 min		Non-MS 40 mg/kg MS MS Model**** 40 mg/kg^####^			
							Nest Building	24 h		Non-MS 40 mg/kg MS MS Model*** 40 mg/kg^####^			
p.o.	Subchronic (3 d)	Water	Rat	Wistar	Male	n = 6–9 per group Control Binge Drinking (EtOH) Model Binge + AEGI	Binge Drinking	35 d	Weekly (daily administration for 3 consecutive days)	n/a	n/a	Models binge drinking induced depression	Nascimento et al. (2020)
							FST	Last 3 min of 5 min	Once	Binge Drinking Model**** 0.1 mL/100 g^##^			
p.o.	Acute (60 min)	Petroleum Ether, Chloroform, Methanol, and Water Methanol → Ethyl Acetate, n-Butanol, and Methanol fractions	Mouse	Swiss Albino	Male	n = 6 per group Control Extracts Pet. Ether (Low, Medium, High) Chloroform (Low, Medium, High) Methanol (Low, Medium, High) Aqueous (Low, Medium, High) Imipramine Fractions **E**: Ethyl Acetate (Very Low, Low, Medium) **N**: n-Butanol (Very Low, Low, Medium) **MF**: Methanol-soluble fraction (Low, Medium, High) Imipramine	FST	Last 4 min of 6 min	Once	Extracts 100 mg/kg* 200 mg/kg* 400 mg/kg* **(for all extracts)** Fractions 50 mg/kg – E*, N* 100 mg/kg – E*, N, MF* 200 mg/kg – E*, N*, MF* 400 mg/kg – MF*	n/a	n/a	Singh et al. (2021)
i.g.	Acute (30 min)	Water	Mouse	Swiss Albino	Male	n = 11–12 per group Control *G*. *lucidum* extract (Very Low, Low, Medium, High)	FST	Last 4 min of 6 min	Once	50 mg/kg 100 mg/kg** 200 mg/kg*** 400 mg/kg***	n/a	Mycelium	Socala et al. (2015)
p.o.	Chronic (28 d)	Water	Mouse	C57BL/6	Male	n = 10 per group Control Control + PGL (Low, Medium, High) UCMS Model UCMS + PGL (Low, Medium. High) UCMS + Fluoxetine	UCMS	56 d	Daily	n/a	n/a	Spore polysaccharide-peptide	Zhao et al. (2021)
							SPT	24 h	Once	UCMS Model** 100 mg/kg^#^ 200 mg/kg^##^ 400 mg/kg^##^			
	Acute (1 h) Chronic (28 d)						FST	Last 4 min of 6 min		Acute 100 mg/kg 200 mg/kg* 400 mg/kg** Chronic UCMS Model 100 mg/kg^##^ 200 mg/kg^##^ 400 mg/kg^##^			
	Acute (1 h)						TST			100 mg/kg 200 mg/kg** 400 mg/kg**			
*Ganoderma applanatum*													
p.o.	Acute (30 min)	Ethanol and Water	Mouse	Swiss Albino	Both	n = 5 per group Control Ethanol (Low, High) Aqueous (Low, High) Diazepam [i.p.]	TST	6 min	Once	Ethanol 200 mg/kg 400 mg/kg Aqueous 200 mg/kg 400 mg/kg	n/a	n/a	Hossen et al. (2021)
p.o.	Acute (60 min)	Petroleum Ether, Chloroform, Methanol, and Water	Mouse	Swiss Albino	Male	n = 6 per group Control Extracts Pet. Ether (Low, Medium, High) Chloroform (Low, Medium, High) Methanol (Low, Medium, High) Aqueous (Low, Medium, High) Imipramine	FST	Last 4 min of 6 min	Once	Extracts 100 mg/kg* 200 mg/kg* 400 mg/kg* **(for all extracts)**	n/a	n/a	Singh et al. (2021)
*Ganoderma philippii*													
p.o.	Acute (60 min)	Petroleum Ether, Chloroform, Methanol, and Water	Mouse	Swiss Albino	Male	n = 6 per group Control Extracts Pet. Ether (Low, Medium, High) Chloroform (Low, Medium, High) Methanol (Low, Medium, High) Aqueous (Low, Medium, High) Imipramine	FST	Last 4 min of 6 min	Once	Extracts 100 mg/kg* 200 mg/kg* 400 mg/kg* **(for all extracts)**	n/a	n/a	Singh et al. (2021)
*Ganoderma brownii*													
p.o.	Acute (60 min)	Petroleum Ether, Chloroform, Methanol, and Water	Mouse	Swiss Albino	Male	n = 6 per group Control Extracts Pet. Ether (Low, Medium, High) Chloroform (Low, Medium, High) Methanol (Low, Medium, High) Aqueous (Low, Medium, High) Imipramine	FST	Last 4 min of 6 min	Once	Extracts 100 mg/kg* 200 mg/kg* 400 mg/kg* **(for all extracts)**	n/a	n/a	Singh et al. (2021)
*Ganoderma sp.*													
i.v.	Chronic (21 d)	n/a	Rat	Sprague-Dawley	Male	Sham (n = 8) MCAO (n = 7) PSD Model (n = 7) PSD + GAA (Low, Medium, High) (n = 8)	PSD (UCMS)	21 d	Daily	n/a	n/a	Ganoderic acid (triterpenoid) Performs MCAO to induce stroke conditions Use UCMS to establish PSD	Zhang et al. (2021a)
							SPT	3 h	Once	PSD Model^^ 10 mg/mL 20 mg/mL^#^ 30 mg/mL^##^ ^^p < 0.01 v.s. MCAO group			
*Grifola frondosa*													
Food	Subchronic: Cohort 1 (5 d) Cohort 2 (1 d) Cohort 3 (5 d)	n/a	Mouse	CD-1	Male	Cohort 1 (n = 14 per group) Cohort 2 (n = 14 per group) Cohort 3 (n = 10–11 per group) For each cohort: Control Low Medium High Imipramine	FST	Last 4 min of 6 min	Once	1:4 GF:chow** 1:2 GF:chow** 1:1 GF:chow***	n/a	Tested with multiple cohorts	Bao et al. (2017)
	Subchronic: Cohort 1 (1 d) Cohort 2 (5 d) Cohort 3 (1 d)					Cohort 1 (n = 14 per group) Cohort 2 (n = 13 per group) Cohort 3 (n = 11 per group) For each cohort: Control Low Medium High Imipramine	TST			1:4 GF:chow* 1:2 GF:chow** 1:1 GF:chow**			
*Psilocybe cubensis*													
p.o. (whole) i.p. (extracts)	Acute (30 min)	Methanol and Water	Mouse	Swiss Webster	Male	n ≥ 7 per group Control Whole Mushroom (Very High) Methanol (Low, Medium, High) Aqueous (Low, Medium, High) Fluoxetine [s.c.] Imipramine [i.p.]	FST	5 min	Once	Whole Mushroom 1000 mg/kg* Methanol 1 mg/kg 10 mg/kg** 100 mg/kg*** Aqueous 1 mg/kg** 10 mg/kg*** 100 mg/kg***	n/a	n/a	Hernandez-Leon et al. (2024)
i.p.	Acute (30 min)	Chloroform	Mouse	NMRI	Male	n = 8 per group Control PCE (Low, High) PCE (Low) + Ketamine PCE (High) + Ketamine Ketamine Fluoxetine	FST	Last 4 min of 6 min	Once	10 mg/kg 40 mg/kg For PCE (10 mg/kg): PCE + Ketamine (1 mg/kg)*** For PCE (40 mg/kg): PCE + Ketamine (1 mg/kg)***	Ketamine	Alkaloid extract	Mahmoudi et al. (2018)
							TST						
*Pleurotus eryngii*													
p.o.	Chronic (84 d)	Ethanol	Rat	Wistar	Female	Sham (n = 10) OVX Model (n = 10) OVX + *P. eryngii* (n = 8)	OVX	84 d	Once	n/a	n/a	Models menopausal depression	Minami et al. (2013)
	Chronic (79 d)						FST	6 min	Once	OVX Model* 500 mg/kg^#^			
i.p.	Acute (30 min)	Ethanol	Mouse	n/a	n/a	n = 4 per group Control EtOH Extract Mixture (pellet) R2 Fraction Fluoxetine	FST	4 min	Once	EtOH Extract* Mixture (pellet)** R2 Fraction* (all 20 mg/kg)	n/a	EtOH Extract → Pellet → R2: fractions increase in purification levels	Park et al. (2021)
*Pleurotus ostreatus*													
Food	Subchronic (5 d)	n/a	Mouse	CD-1	Male	Control (n = 11) PO (n = 11) Imipramine (n = 10–11)	FST	Last 4 min of 6 min	Once	1:2 PO:chow	n/a	n/a	Bao et al. (2017)
	Subchronic (1 d)						TST						
*Pleurotus citrinopileatus*													
Food	Chronic (21 d)	n/a	Mouse	C57BL/6J	Male	Control (n = 6) Control + 10% (n = 8) CRS Model (n = 8) CRS + 10% (n = 8)	CRS	21 d	4 h daily	n/a	n/a	Antioxidant ergothioneine (ERGO) and golden oyster mushroom extract (GOME)	Nakamichi et al. (2016)
	Chronic (14 d)					Control (n = 11) 10% GOME (n = 11) ERGO (n = 6) *Ginkgo biloba* extract (n = 6)	FST	5 min	Once	10% GOME* 120 mg/100 g ERGO*			
						Control (n = 15) 0.1% GOME (n = 6) 0.3% GOME (n = 6) 1% GOME (n = 12) 10% GOME (n = 15)	TST	First 2 min of 3 min		0.1% 0.3% 1%* 10%*			
*Marasmius androsaceus*													
p.o.	Subchronic (7 d)	n/a	Mouse	Kunming	Male	n = 8 per group Control MEPS1 (High) MEPS2 (Medium) MEPS3 (Low)	FST	6 min	Once	180 mg/kg 60 mg/kg* 30 mg/kg	n/a	Extracellular polysaccharide	Song et al. (2020)
							TST	5 min		180 mg/kg* 60 mg/kg** 30 mg/kg			
p.o.	Chronic (28 d)	n/a	Rat	Sprague-Dawley	Male	n = 10 per group Control UCMS Model UCMS + MEPS (Low, Medium, High)	UCMS	56 d	Daily	n/a	n/a	Exopolysaccharides	Song et al. (2017)
							SPT	1 h	Weekly for 7 weeks	UCMS Model** 6 mg/kg 30 mg/kg^#^ 150 mg/kg^##^ *Model: significant from day 14 to day 56* *MEPS: 30 mg/kg and 150 mgm/kg significant from day 49 to day 56*			
							FST	Last 5 min of 6 min	Once	UCMS Model*** 6 mg/kg^#^ 30 mg/kg^###^ 150 mg/kg^###^			
							TST			UCMS Model*** 6 mg/kg 30 mg/kg^###^ 150 mg/kg^###^			
p.o.	Subchronic (7 d)	n/a	Mouse	Kunming	Both	n = 10 per group Control MEPS (Low, Medium, High) Fluoxetine [i.g.]	FST	Last 5 min of 6 min	Once	10 mg/kg 50 mg/kg 250 mg/kg*	n/a	Exopolysaccharide	Song et al. (2016)
							TST	6 min		10 mg/kg 50 mg/kg* 250 mg/kg***			
i.g.	Chronic (14 d)	n/a	Mouse	C57BL/6J	Male	n = 8 per group IR CRS Model CRS + M	IR + Dp (CRS)	21 d	4 h daily	n/a	n/a	Mycelium Mice were irradiated with 13 Gy TAI to induce intestinal radiation injury CRS was used to induce depression	Zhao et al. (2023)
							FST	Last 4 min of 6 min	Once	CRS Model^+++^ CRS + M^##^ ^+++^p < 0.001 v.s. IR group			
							TST			CRS Model^++^ CRS + M^##^ ^++^p < 0.01 v.s. IR group			
*Collybia maculata*													
i.p.	Acute (immediate)	n/a	Mouse	C57BL/6J	Male	n = 7–10 per group Vehicle Colly	FST	6 min	Once	2 mg/kg	n/a	Colly: non-nitrogenous sesquiterpene of *C. maculata*	Gupta et al. (2016)
*Poria cocos*													
p.o.	Chronic (35 d)	Water	Rat	Sprague-Dawley	Male	n = 7 per group Control Control + PCW (Low, Medium, High) UCMS Model UCMS + PCW (Low, Medium, High)	UCMS	35 d	Daily	n/a	n/a	Sclerotium	Huang et al. (2020)
	Chronic (35 d)						SPT	1 h	Weekly for 5 weeks	UCMS Model* 100 mg/kg^#^ 300 mg/kg^#^ 900 mg/kg (*After 4 weeks*)			
	Chronic (28 d)						FST	5 min	Once	100 mg/kg* 300 mg/kg* 900 mg/kg*			
*Lentinula edodes*													
p.o.	Acute (2 h) Chronic (14 d)	n/a	Mouse	ICR	Male	n = 5 per group Control Pilopool	FST	Last 4 min of 6 min	Once	Acute 10 mL/kg* Chronic 10 mL/kg	Pilopool mixture: 30% of *L. edodes*/shiitake extract + 30% water-soluble chitosan, 30% *Allium sativum L.* extract, 0.5% of *Dioscorea batatas D*., and 0.5% of bamboo salt	n/a	Koo et al. (2008)
*Armillaria mellea*													
p.o.	Chronic (35 d)	Water	Rat	Sprague-Dawley	Male	n = 7 per group Control UCMS Model UCMS + WAM (Low, Medium, High) UCMS + Fluoxetine	UCMS	35 d	Daily	n/a	n/a	n/a	Lin et al. (2021a)
	Chronic (34 d)						SPT	1 h	Once	UCMS Model** 250 mg/kg^#^ 500 mg/kg^#^ 1000 mg/kg^#^			
	Chronic (30 d)						FST	5 min		250 mg/kg^####^ 500 mg/kg^####^ 1000 mg/kg^####^			
i.p.	Acute (30 min)	Ethyl Acetate	Mouse	ICR	Male	n = 10 per group Control PSAM (Lowest, Very Low, Low, Medium, High, Very High, Highest) Fluoxetine	FST	Last 4 min of 6 min	Once	0.05 mg/kg 0.1 mg/kg 0.5 mg/kg* 1 mg/kg* 5 mg/kg* 20 mg/kg 50 mg/kg	n/a	Protoilludane sesquiterpenoid aromatic esters	Zhang et al. (2021b)
							TST			0.05 mg/kg 0.1 mg/kg 0.5 mg/kg* 1 mg/kg** 5 mg/kg* 20 mg/kg* 50 mg/kg For PSAM (0.1 mg/kg): PSAM + Fluoxetine (5 mg/kg)* PSAM + Reboxetine (2.5 mg/kg)**	Fluoxetine Reboxetine		
*Agaricus brasiliensis*													
p.o.	Chronic (30 d)	Water	Mouse	Kunming	Male	n = 10 per group Control UCMS Model UCMS + AWE	UCMS	28 d	Daily	n/a	n/a	n/a	Xin et al. (2022)
							TST	Last 5 min of 6 min	Once	UCMS Model* 3 g/kg^#^			
*Xylaria sp.*													
i.g.	Chronic (28 d)	n/a	Rat	Sprague-Dawley	Male	n = 6–9 per group Control UCMS Model UCMS + Wuling powder (Low, Medium, High) UCMS + Fluoxetine	UCMS	42 d	Daily	n/a	n/a	Wuling mycelia powder	Tan et al. (2016)
							SPT	1 h	Weekly for 6 weeks	UCMS Model*** 0.5 g/kg^#^ 1 g/kg^###^ 2 g/kg^###^ *Model: significant from week 2 to week 6* *Wuling: significant from week 6*			
*Antrodia cinnamomea*													
p.o.	Chronic (16 d)	n/a	Mouse	Kunming	Both	n = 24 per group Control AC (Low, Medium, High)	Weight-loaded FST	n/a	Once	0.1 g/kg 0.3 g/kg** 0.9 g/kg**	n/a	Mycelium Does not focus on depression nor use valid screen	Liu et al. (2017b)
*Mushrooms* (General)													
i.p.	Acute (60 min)	n/a	Mouse	ICR	Male	n = 10 per group Er, ErF, ErS, ErN (Low, High) ErN (Very Low, Low, Medium, High) Fluoxetine Er (Low) + Fluoxetine Er (Low) + Tianeptine Er (Low) + Reboxetine	FST	Last 4 min of 6 min	Once	All derivatives: 5 mg/kg – Er*, ErF*, ErS*, ErN** 20 mg/kg – Er*, ErF*, ErS*, ErN** ErN: 0.1 mg/kg* 0.5 mg/kg 1 mg/kg* 5 mg/kg** For ErN (0.5 mg/kg): ErN + Fluoxetine (5 m/g/kg) ErN + Tianeptine (15 mg/kg)** ErN + Reboxetine (2.5 mg/kg)**	Fluoxetine Tianeptine Reboxetine	Ergosterol and derivatives	Lin et al. (2017)
i.g.	Subchronic (1 d) *Injected 3 times (23.5 h, 5 h, and 1 h prior to test)*	n/a	Rat	Long Evans	Male	n = 10 per group Control Psilocybin Baeocystin Norbaeocystin Aeruginascin Fluoxetine	FST	5 min	Once	Psilocybin* Baeocystin Norbaeocystin* Aeruginascin (all 1 mg/kg)	n/a	Baeocystin, norbaeocystin, aeruginascin: tryptamine alkaloids and analogs of psilocybin Preprint; not peer-reviewed	Rakoczy et al. (2023)
s.c.	Subchronic (3 d)	n/a	Mouse	ICR	Male	n = 10 per group Control CORT Model CORT + p-CA	CORT	23 d	Daily	20 mg/kg	n/a	P-Coumaric acid (p-CA)	Yu et al. (2022)
i.p.							SPT	24 h	Once	CORT Model*** 75 mg/kg^###^			
							FST	Last 4 min of 6 min		CORT Model* 75 mg/kg^#^			

*p < 0.05, **p < 0.01, ***p <0.001, ****p < 0.0001 compared to control.

^#^ *p* < 0.05, ^##^ *p* < 0.01, ^###^ *p* < 0.001, ^####^ *p* < 0.0001 compared to model/vehicle.

Acute (< 1 d), Subchronic (1–7 d), Chronic (> 7 d).

Abbreviations: FST = forced swim test; TST = tail suspension test; OVX = ovariectomy; UCMS = unpredictable chronic mild stress; CORT = corticosterone; SPT = sucrose preference test; CRS = chronic restraint stress; CSDS = chronic social defeat stress; PSD = post-stroke depression; MS = maternal separation; LPS = lipopolysaccharide; MCAO = middle cerebral artery occlusion; HE = *Hericium erinaceus;* Gl-E = *Ganoderma lucidum* extract; EEGL = ethanol extract of *Ganoderma lucidum;* GLP = *Ganoderma lucidum* polysaccharide; MAK = *Ganoderma lucidum* mycelia; GLT = *Ganoderma lucidum* triterpenoid; AEGI = aqueous extract of *Ganoderma lucidum;* PGL = Polysaccharide-peptide of *Ganoderma lucidum;* GAA = Ganoderic acid; PCE = *Psilocybe cubensis* extract; PO = *Pleurotus ostreatus;* EtOH = ethanol; MEPS = exopolysaccharide polysaccharide of *Marasmius androsaceus;* PCW = *Poria cocos* water extract; WAM = water extract of *Armillaria mellea;* PSAM = Protoilludane sesquiterpenoid aromatic esters from *Armillaria mellea;* AWE = *Agaricus brasiliensis* water extract; AC = *Antrodia cinnamomea;* Er = Ergosterol; IR = intestinal radiation; E_2_ = 17*β*-estradiol; Dp = depression; i.p. = intraperitoneal; p.o. = per os (oral); i.g. = intragastric; s.c. = subcutaneous; i.v. = intravenous.

**TABLE 2:** Summary of rodent depression models and behavioural tests used to screen for antidepressant effects in different non-mushroom species of fungi. Subchronic and chronic treatment schedules include daily administration of drug unless otherwise stated.

*Cordyceps militaris*													
p.o.	Chronic (34 d)	Water	Rat	Sprague-Dawley	Male	n = 6 per group Control UCMS Model UCMS + CW (Low, Medium, High) UCMS + Fluoxetine	UCMS	34 d	Daily	n/a	n/a	n/a	Lin et al. (2021b)
							SPT	1 h	Once	UCMS Model*** 125 mg/kg^###^ 250 mg/kg^#^ 500 mg/kg^#^			
i.g.	Chronic (42 d)	n/a	Mouse	ICR	Male	n = 20 per group Control UCMS Model UCMS + COR (Low, High) UCMS + Fluoxetine	UCMS	42 d	Daily	n/a	n/a	Cordycepin (3'-deoxyadenosine): component of *C. militaris*	Tianzhu et al. (2014)
							SPT	12 h	Twice (Weeks 3 and 6)	Week 3 UCMS Model 20 mg/kg 40 mg/kg Week 6 UCMS Model** 20 mg/kg^##^ 40 mg/kg^##^			
							FST	Last 4 min of 6 min	Once	UCMS Model** 20 mg/kg^#^ 40 mg/kg^##^			
							TST		Twice (Weeks 3 and 6)	Week 3 UCMS Model 20 mg/kg 40 mg/kg Week 6 UCMS Model** 20 mg/kg^##^ 40 mg/kg^##^			
i.g.	Chronic (28 d)	Water	Mouse	Kunming	Male	n = 12 per group Control PCM (Low, Medium, High)	Weight-loaded FST	n/a	Once	40 mg/kg* 80 mg/kg* 160 mg/kg*	n/a	Polysaccharide Does not focus on depression nor use valid screen	Xu (2016)
*Cordyceps sinensis*													
p.o.	Subchronic (5 d)	Supercritical Fluid and Hot Water	Mouse	C57BL/6	Male	n = 17 per group Control Supercritical (Low, Medium, High) Aqueous (Low, Medium, High)	TST	6 min	Once	Supercritical 2.5 mL/kg 5 mL/kg* 10 mL/kg* Aqueous 500 mg/kg 1000 mg/kg 2000 mg/kg	n/a	n/a	Nishizawa et al. (2007)
p.o.	Chronic (30 d)	n/a	Mouse	Swiss Albino	Both	n = 6 per group Control Natural *C. sinensis* (Low, Medium, High) Lab-cultured Mycelia (Low, Medium, High) Fluoxetine	Photoactometer	n/a	Once	NC 100 mg/kg 300 mg/kg* 500 mg/kg* LCM 100 mg/kg 300 mg/kg* 500 mg/kg*	n/a	Mycelium	Singh et al. (2014)
*Paecilomyces tenuipes*													
p.o.	Chronic (28 d)	Water	Rat	Sprague-Dawley	Male	n = 10 per group Control UCMS Model UCMS + PTNE (Low, Medium, High) UCMS + Fluoxetine	UCMS	56 d	Daily	n/a	n/a	Cultured mycelium	Liu et al. (2017a)
							FST	Last 5 min of 6 min	Once	UCMS Model*** 0.04 g/kg^##^ 0.2 g/kg^###^ 1 g/kg^###^			
p.o.	Chronic (21 d)	Alcohol and Water	Mouse	n/a	Male	n = 10 per group Control Control + AE (Low, Medium, High) Control + WE (Low, Medium, High) Control + Fluoxetine UCMS Model UCMS + AE (Low, Medium, High) UCMS + WE (Low, Medium, High) UCMS + Fluoxetine	UCMS	21 d	Daily	n/a	n/a	Mutant *P. tenuipes* strain M98 Mycelium	Li et al. (2019)
							SPT	1 h	Once	**UCMS** UCMS Model* Alcohol 0.05 g/kg 0.25 g/kg 2.5 g/kg^#^ Water 0.04 g/kg 0.2 g/kg 2 g/kg^#^			
	Chronic (15 d), Chronic (21 d; UCMS)						FST	Last 4 min of 6 min		**Non-UCMS** Alcohol 0.05 g/kg 0.25 g/kg 2.5 g/kg* Water 0.04 g/kg 0.2 g/kg 2 g/kg** **UCMS** UCMS Model* Alcohol 0.05 g/kg 0.25 g/kg 2.5 g/kg^#^ Water 0.04 g/kg^#^ 0.2 g/kg 2 g/kg^#^			
							TST			**Non-UCMS** Alcohol 0.05 g/kg* 0.25 g/kg 2.5 g/kg Water 0.04 g/kg* 0.2 g/kg 2 g/kg* **UCMS** UCMS Model* Alcohol 0.05 g/kg 0.25 g/kg 2.5 g/kg Water 0.04 g/kg 0.2 g/kg^#^ 2 g/kg^#^			
*Paecilomyces hepiali*													
p.o.	Chronic (28 d)	Water	Rat	Sprague-Dawley	Male	n = 6 per group Control UCMS Model UCMS + PHC (Low, Medium, High) UCMS + Fluoxetine	UCMS	56 d	Daily	n/a	n/a	n/a	Wang et al. (2017)
							SPT	2 h	Once	UCMS Model** 0.08 g/kg 0.4 g/kg^#^ 2 g/kg^##^			
							FST	Last 5 min of 6 min		UCMS Model* 0.08 g/kg^#^ 0.4 g/kg 2 g/kg^##^			
*Ophiocordyceps formosana*													
i.p.	Subchronic (5 d)	n/a	Mouse	C57BL/6	Male	Control (n = 6) STZ Model (n = 8) STZ + OFE (n = 8) STZ + Rosiglitazone (n = 8)	STZ	5 d	Daily	40 mg/kg	n/a	Uses STZ to induce diabetes Models diabetes-induced depression	Huang et al. (2016)
p.o.	Chronic (28 d)						TST	6 min	Once	STZ Model* 25 mg/mL^#^			
*Penicillium sp.*													
i.p.	Acute (30 min)	n/a	Mouse	ICR	Male	n = 8 per group 36 groups Control 2a–2i 3a–3r 4a–4g Fluoxetine	FST	Last 4 min of 6 min	Once	0.1 mL/20 g* *28 compounds showed significant antidepressant effect (26.23% – 89.96% decrease in immobility time vs. control)	n/a	Compounds are derivatives of *P. sp.*	Jin et al. (2019)
*Beauveria sp.*													
i.g.	Chronic (21 d)	n/a	Mouse	Kunming	Male	n = 10 per group Control UCMS Model UCMS + BCEF (Low, Medium, High) UCMS + Moclobemide	UCMS	21 d	Daily	n/a	n/a	BCEF0083: bioactive compound	Zhou et al. (2005)
							SPT	24 h	Once	UCMS Model** 25 mg/kg^##^ 50 mg/kg^##^ 100 mg/kg^##^			

* p < 0.05, **p < 0.01, ***p <0.001, ****p < 0.0001 compared to control

^#^ p < 0.05, ^##^p < 0.01, ^###^p < 0.001, ^####^p < 0.0001 compared to model/vehicle

Acute (< 1 d), Subchronic (1–7 d), Chronic (> 7 d)

Abbreviations: FST = forced swim test; TST, tail suspension test; UCMS, unpredictable chronic mild stress; SPT, sucrose preference test; STZ, streptozotocin(-induced diabetes); CW, *Cordyceps militaris* water extract; COR, Cordycepin; PCM = polysaccharide of *Cordyceps militaris;* PTNE = *Paecilomyces tenuipes* N45 aqueous extract; AE, alcohol extract; WE, water extract; PHC, *Paecilomyces hepiali* extract; OFE, *Ophiocordyceps formosana* extract; BCEF = bioactive compound from entomogenous fungi; i.p. = intraperitoneal; p.o. = per os (oral); i.g. = intragastric.

### Characteristics of animals used and drug administration

The species used in all of the animal models were limited to rats and mice: we did not find instances of other rodent species that have been utilized as antidepressant screens (Kramer et al., 1998; Alo et al., 2019). Fourteen of the studies used rats (Matsuzaki et al., 2013; Minami et al., 2013; Tan et al., 2016; Liu C. et al., 2017; Song et al., 2017; Wang et al., 2017; Huang et al., 2020; Nascimento et al., 2020; Lin et al., 2021a; Zhang L. et al., 2021; Lin et al., 2021b; Anuar et al., 2022; Cheng, 2023; Rakoczy et al., 2023), while the remaining 36 studies used mice to test for antidepressant-like effects (Zhou et al., 2005; Nishizawa et al., 2007; Koo et al., 2008; Singh et al., 2014; Tianzhu et al., 2014; Socala et al., 2015; Yao et al., 2015; Gupta et al., 2016; Huang et al., 2016; Nakamichi et al., 2016; Song et al., 2016; Xu, 2016; Bao et al., 2017; Liu Y. et al., 2017; Lin et al., 2017; Chiu et al., 2018; Mahmoudi et al., 2018; Ryu et al., 2018; Jin et al., 2019; Li et al., 2019; Song et al., 2020; Li H. et al., 2021; Li TJ. et al., 2021; Zhang T. et al., 2021; Chong et al., 2021; Hossen et al., 2021; Park et al., 2021; Singh et al., 2021; Zhao et al., 2021; Chou et al., 2022; Mi et al., 2022; Xin et al., 2022; Yu et al., 2022; Ezurike et al., 2023; Zhao et al., 2023; Hernandez-Leon et al., 2024). In terms of strains, 10 of the rat studies used Sprague-Dawley, three included Wistar and one used Long-Evans rats. For the mice studies, the most commonly used strain was the C57BL/6 strain and sub-strains (12 studies), followed by Institute for Cancer Research mice (seven studies), Swiss Albino and Kunming mice (six studies each) and one study each with BALB/c, CD1, Swiss Webster and NMRI strains. Two studies did not mention the specific strains used (Li et al., 2019; Park et al., 2021). The overwhelming majority of rat studies used male rats (12 studies) compared to female rats (two studies (Minami et al., 2013; Anuar et al., 2022)). All mice studies utilized males, most of which included males only, while five studies used both male and female mice (Singh et al., 2014; Song et al., 2016; Liu Y. et al., 2017; Hossen et al., 2021; Ezurike et al., 2023), and one study did not specify sex (Park et al., 2021). Thus, only 14% of studies used female animals in their investigation.

Administration of mushroom or fungus derivatives to animals was mostly through a single route of administration, although a handful of studies used two different routes of administration (Yao et al., 2015; Huang et al., 2016; Chou et al., 2022; Yu et al., 2022; Hernandez-Leon et al., 2024). The most common route of administration was oral (*per os*, p.o.), which accounted for more than 50% of studies (29 of 55 instances of administration). Second most common was treatment by intraperitoneal (i.p.) injection (13 instances), followed by intragastric (i.g.) administration (7 instances). Extracts were administered to animals in their food in three separate studies (Nakamichi et al., 2016; Bao et al., 2017; Anuar et al., 2022), by subcutaneous (s.c.) injection in two studies (Chou et al., 2022; Yu et al., 2022), and by intravenous (i.v.) administration in one study (Zhang L. et al., 2021).

The methods of extraction of mushroom and fungus derivatives was reported in 28 studies. Methods included use of both polar and non-polar solvents, with the most common ones including water and various alcohols. For many of the studies where complex extraction procedures were involved, including with non-polar solvents, it was not possible to determine if the extracts that were administered to animals also contained traces of these solvents (e.g., (Singh et al., 2021)), which could feasibly have an effect on behaviour.

The duration of drug treatment varied significantly across the studies, from acute doses with behavioural testing 30 min later (Socala et al., 2015; Mahmoudi et al., 2018; Jin et al., 2019; Zhang T. et al., 2021; Hossen et al., 2021; Park et al., 2021; Hernandez-Leon et al., 2024), up to 92 days of continuous administration (Anuar et al., 2022). Of the 50 studies, 13 were acute (treatment over a span of <24 h) (Matsuzaki et al., 2013; Socala et al., 2015; Yao et al., 2015; Gupta et al., 2016; Lin et al., 2017; Mahmoudi et al., 2018; Jin et al., 2019; Zhang T. et al., 2021; Hossen et al., 2021; Park et al., 2021; Singh et al., 2021; Rakoczy et al., 2023; Hernandez-Leon et al., 2024), six were sub-acute (1–7 days) (Nishizawa et al., 2007; Song et al., 2016; Bao et al., 2017; Nascimento et al., 2020; Song et al., 2020; Yu et al., 2022), and the remaining 31 studies involved chronic treatment (>7 days). The mean duration of treatment for the chronic studies was 30.4 (±16.7) days for the longest treated group in each study (some studies had varying durations of treatment depending on the group). The modal and median periods of treatment for chronic studies were both 28 days. Rats were more likely to be treated chronically, with only two of the 14 rat studies involving acute treatment (Matsuzaki et al., 2013; Rakoczy et al., 2023).

### Animal models of depression and tests of antidepressant activity

A variety of animal models of depression and antidepressant screens were used to examine mushroom and fungus antidepressant efficacy. By far the most common animal model used to induce a depressive-like phenotype in rodents was the unpredictable chronic mild stress paradigm (UCMS), with 14 studies implementing this model (Zhou et al., 2005; Tianzhu et al., 2014; Tan et al., 2016; Liu C. et al., 2017; Song et al., 2017; Wang et al., 2017; Li et al., 2019; Huang et al., 2020; Lin et al., 2021a; Zhang L. et al., 2021; Lin et al., 2021b; Zhao et al., 2021; Xin et al., 2022; Cheng, 2023); rats were used in the majority (9) of these studies. The second most frequent model involved the use of chronic restraint stress, in four mouse studies (Nakamichi et al., 2016; Chiu et al., 2018; Chong et al., 2021; Zhao et al., 2023). Two rat studies used ovariectomy procedures to model menopausal depression (Minami et al., 2013; Anuar et al., 2022), while high-dose corticosterone was administered to mice in two studies (Chou et al., 2022; Yu et al., 2022). Other models included the use of lipopolysaccharide (Yao et al., 2015), chronic social defeat stress (Li H. et al., 2021), maternal separation (Mi et al., 2022), ethanol binge drinking (Nascimento et al., 2020) and streptozotocin to model diabetes-induced depression (Huang et al., 2016). To determine that a depressive-like state had been induced by the animal models, which could then be reversed by compounds with antidepressant activity, behavior was predominantly assessed with three main tests, which included the forced swim test (FST) (19 studies), tail suspension test (TST) (13 studies) and sucrose preference test (SPT) (16 studies)—multiple studies used two or more of these tasks. One study assessed behavior in the splash test as well as nest building (Mi et al., 2022), while one study measured locomotor activity and neuromuscular endurance (Singh et al., 2014). Twenty one of the 50 studies did not use an animal model of depression *per se*, and tested antidepressant activity solely with standalone antidepressant screens. This included 18 studies which used the FST and 10 that used the TST (seven studies used both); only two of these 21 studies used rats (Matsuzaki et al., 2013; Rakoczy et al., 2023).

#### Antidepressant effects of mushroom extracts

The Kingdom Fungi encompasses many known species which can be further classified into subgroups by the mechanism with which they reproduce and disseminate their spores (Boundless, 2024). Fungi subcategories include mushrooms, as well as other fungi such as moulds and yeasts. Mushrooms from the genus *Psilocybe* are of particular interest as many from the genus are known to contain the psychoactive compounds psilocybin and psilocin. This includes the species *Psilocybe cubensis*, which has been demonstrated to be able to alleviate depression and anxiety symptoms in clinical trials (Ross et al., 2016; Goodwin et al., 2022). Other mushrooms species such as *H. erinaceus* and *G. lucidum* do not necessarily contain psychoactive compounds, but are still of interest in models and studies of depression. Most research investigating the use of medicinal mushrooms and their extracts to treat depression has been in preclinical settings, rather than in clinical trials.

Of the 19 species of mushroom tested for antidepressant-like activity in the current review, the most common one was *G. lucidum*, in nine studies Table 1. Two studies used UCMS and reported 28-day treatment with doses of 100–500 mg/kg, p.o. exerted antidepressant-like effects in the SPT (Cheng, 2023) and both the SPT and FST (Zhao et al., 2021). A 5 mg/kg, i.p. dose in mice exerted antidepressant-like effects in the TST and FST after chronic social defeat stress (Li H. et al., 2021), while effects in mice subjected to the maternal separation model were reversed with a 21-day treatment with 40 mg/kg, i.p. of extract (Mi et al., 2022); 100 mg/kg, p.o. also reversed immobility in the FST in a binge-alcohol model (Nascimento et al., 2020). Antidepressant screens found positive effects with chronic doses of 100–1,000 mg/kg, p.o. in the FST and TST (Matsuzaki et al., 2013; Socala et al., 2015; Singh et al., 2021; Ezurike et al., 2023). Significant antidepressant-like effects were observed with the UCMS model with *Ganoderma* sp. extracts (21-day, 20–30 mg/kg, i.v.) (Zhang L. et al., 2021); in this study, the authors did not specify with species of *Ganoderma* the active compound ganoderic acid-a was extracted from.

*Hericium erinaceus* was examined in seven studies. Extracts (25 mg/kg, i.p. and 200–400 mg/kg, p.o.) for 28 days reversed the effects of chronic restraint stress in the SPT, TST (Chong et al., 2021) and FST (Chiu et al., 2018). Doses of 12–24 mg (combined with *Chlorella Vulgaris*), p.o. for 21 days significantly reversed immobility in the FST caused by treatment with high dose corticosterone (Chou et al., 2022). A single oral dose of 200 mg/kg reversed increased immobility in the FST and TST caused by lipopolysaccharide (Yao et al., 2015), while 28-day administration at 20–60 mg/kg, p.o. decreased immobility in the TST and FST (Ryu et al., 2018).

For other mushroom species examined, effects were observed with the UCMS model with *Marasmius androsaceus* (28-day, 30–150 mg/kg, p.o.), *Poria cocos* (35-day, 100–300 mg/kg, p.o.) (Huang et al., 2020), *Armillaria mellea* (35-day, 250–1,000 mg/kg, p.o.) (Lin et al., 2021a), *Agaricus brasiliensis* (30-day, 3,000 mg/kg, p.o.) (Xin et al., 2022) and *Xylaria sp*. (28-day, 500–2000 mg/kg, i.g.) (Tan et al., 2016). Other animal models included antidepressant-like effects in a model of menopausal depression (*Pleurotus eryngii*, 79-day, 500 mg/kg, p.o.) (Minami et al., 2013), chronic restraint stress (*Pleurotus citrinopileatus,* 14-day, 1,200 mg/kg, in food) (Nakamichi et al., 2016) (*M. androsaceus*, 14-day, i.g.) (Zhao et al., 2023) and high-dose corticosterone (P-coumaric acid–compound found in some mushrooms, 3-day, 75 mg/kg, i.p.) (Yu et al., 2022).

As an antidepressant screen, studies using the standalone FST and TST reported significant antidepressant-like effects with *Ganoderma applanatum*, *Ganoderma philippii,* and *Ganoderma brownii* (single dose, 100–400 mg/kg, p.o.) (Singh et al., 2021), *Grifola frondosa* (1/5-days, in a 1:1-1:4 ratio of *Griflola frondosa* powder to rat chow ratio) while *Pleurotus ostreatus* had no effect in the same study (Bao et al., 2017), *P. cubensis* (single dose. 1,000 mg/kg, p.o. (Hernandez-Leon et al., 2024), and single dose 10–40 mg/kg, i.p., combined with ketamine) (Mahmoudi et al., 2018), *P. eryngii* (single dose, 20 mg/kg, i.p.) (Park et al., 2021), *M. androsaceus* (7-day, 50–250 mg/kg, p.o.) (Song et al., 2016; Song et al., 2020), *Lentinula edodes* (single dose 10 ml/kg p.o., [30% water soluble chitosan, 30% *Allium sativum* extract, 30% *L. edodes* extract, 0.5% Dioscorea Batatas extract, 0.5% bamboo salt extract]) (Koo et al., 2008), *A. mellea* (single dose, 5–20 mg/kg, i.p.) (Zhang T. et al., 2021), as well as ergosterol and derivatives (single dose, 0.1–20 mg/kg, i.p.) (Lin et al., 2017), and the mushroom extracts psilocybin and norbaeocystin (three doses over 24 h, 1 mg/kg, i.g.) (Rakoczy et al., 2023). No antidepressant effect was observed for Collybolide (a fungal metabolite; 2 mg/kg, i.p.) extract (Gupta et al., 2016).

#### Antidepressant effects of fungus extracts

For the seven species of fungus that do not produce mushrooms, antidepressant activity was examined using the UCMS model in six studies Table 2. Antidepressant-like effects on the SPT and/or FST were observed with *Cordyceps militaris* (34-day, 125–500 mg/kg, p.o.) (Lin et al., 2021b) and (42-day, 20–40 mg/kg, i.g.) (Tianzhu et al., 2014), *Paecilomyces tenuipes* (28-day, 40–1,000 mg/kg, p.o.) (Liu C. et al., 2017) and (21-day, 40–2,500 mg/kg, p.o.) (Li et al., 2019), *Paecilomyces hepiali* (28-day, 80–2000 mg/kg, p.o.) (Wang et al., 2017) and *Beauveria* sp. (21-day, 25–100 mg/kg, i.g.) (Zhou et al., 2005). Treatment with *Ophiocordyceps formosana* (28-day, 2.5 mg, p.o.) reversed TST deficits in a streptozotocin-induced model of diabetic depression (Huang et al., 2016). Three studies used standalone animal antidepressant screens, in which *Cordyceps sinensis* decreased immobility in the TST (5-day, 5–10 ml/kg, p.o.) (Nishizawa et al., 2007) and locomotor activity (30-day, 300–500 mg/kg, p.o.) (Singh et al., 2014), while a wide range of *Penicillium sp*. derivatives (single dose, 30 mg/kg, i.p.) were active in the FST (Jin et al., 2019).

## Discussion

In the current analysis, we have summarized the main findings from a scoping review of the effects of mushroom and fungus extracts in preclinical tests of antidepressant efficacy. While this topic covers a broad range of compounds and techniques, several important themes are evident. Firstly, a large number of different species exhibit antidepressant-like activity, including 19 species of mushrooms and seven species of other fungi. For each of these, there can be multiple derivatives with their own antidepressant-like effects; for example, one study with *Penicillium sp*. identified 28 individual compounds with antidepressant-like effects in the FST (Jin et al., 2019), including some with more potent effects than the positive control fluoxetine. Thus, it appears that there is significant potential for novel compounds with antidepressant activity within these organisms. While this includes mushrooms with extracts that have traditionally been associated with psychoactive properties, such as *P. cubensis*, other novel compounds were identified with antidepressant-like effects. For example, P-coumaric acid was found to exhibit antidepressant-like effects after high dose corticosterone treatment (Yu et al., 2022); and was previously reported to exert pro-cognitive and anxiolytic effects in rodents (Scheepens et al., 2014; Kim et al., 2017; Ghaderi et al., 2022). Several of the species evaluated in this review have been tested in humans, confirming benefits for clinical depression. The antidepressant effects of psilocybin and psilocin, which are present in multiple of the current mushroom species are now well established (Griffiths et al., 2016; Ross et al., 2016; Davis et al., 2021; Eisenstein, 2022; Goodwin et al., 2022). In addition, one study showed that menopausal women experienced a reduction in depression and anxiety after 4 weeks of Hericium erinaceus intake (Nagano et al., 2010) while another showed a non-significant trend of reduced depression in women with fibromyalgia who received micromilled *G. lucidum* carpophores for 6 weeks (Pazzi et al., 2020).

Secondly, viewed as a whole, there are a number of both strengths and limitations within this literature. A positive is that the majority of studies administered compounds orally. While for many, use of oral gavage on a daily basis is technically more challenging than i.p. or s.c. drug administration in rodents (Turner et al., 2011), it strongly increases the translational validity of the studies, as human trials will be likely to use the same route of administration and be affected by similar pharmacokinetic processes, such as first-pass metabolism and low bioavailability (Bicker et al., 2020). It is also promising that antidepressant-like effects were observed across a wide duration of treatments with psychedelic and non-psychedelic-containing mushrooms and other fungi. Psychedelic compounds generally induce rapid drug tolerance upon repeated administration (Baumeister et al., 2014; Huang et al., 2022), where 5-HT2A receptor desensitization and/or downregulation leads to functional tolerance that can last several days (Buchborn et al., 2015; de la Fuente Revenga et al., 2022). However, observations of antidepressant-like effects weeks after treatment indicate that therapeutic effects may be sustained with these compounds (Aleksandrova and Phillips, 2021; Kelmendi et al., 2022). Various psychedelics have been reported to enhance neuroplasticity (synapto- and dendritogenesis) in frontocorticolimbic circuitry and increase functional connectivity in the brain, presumably reversing structural and functional deficits in depression (Aleksandrova and Phillips, 2021; Kelmendi et al., 2022). These psychedelic-induced structural and functional changes have been shown to last for weeks to months in animal models and/or humans and are thought to underlie the sustained therapeutic efficacy of these compounds (Aleksandrova and Phillips, 2021; Kelmendi et al., 2022).

While not necessarily a weakness, an extremely wide range of doses of extracts were tested in the current studies. From Tables 1, 2, these range from 1 mg/kg (Li H. et al., 2021; Rakoczy et al., 2023) to 3,000 mg/kg (Xin et al., 2022). Part of this reflects the effects of different routes of administration. Most of the extracts were administered orally, which is associated with a need for higher dosing, and therefore many of these studies included doses in the hundreds of milligrams per kilogram. But this wide range of dosing also represents the likelihood that many of the extracts were in early stage development, where the active compounds are unknown, and so whole product, heterogeneous extracts are used where the efficacy of active compounds may be modified through both pharmacodynamic (e.g., receptor antagonism) and pharmacokinetic (e.g., absorption) processes by many inactive compounds. Thus, such studies are early-stage screens as part of an iterative process (Reis et al., 2017), and in the case of positive effects in the antidepressant screen, this will lead to refinement of extracts by further chemical analysis and result in greater potency, with a lower dose needed.

Multiple different animal models of depression and antidepressant screens were used to test for antidepressant-like effects. Although there is no universally accepted definition, animal models of depression are typically more complex and chronic than antidepressant screens, and are used to emulate some feature(s) of depression, such as its symptoms (face validity) or underlying biology (construct validity) (Geyer et al., 1995; Willner, 1984; Belzung and Lemoine, 2011; van den Berg, 2022). By contrast, antidepressant screens such as the TST and FST are acute and were originally designed to identify novel antidepressant compounds (predictive validity) without regard for similarity to the human condition (Commons et al., 2017). The most commonly used animal model of depression in the present studies was the UCMS paradigm, which is based on the development of anhedonia following exposure to chronic, variable stressors (Willner, 2017; Nollet, 2021). The model has strong theoretical appeal, based on the chronic onset of the antidepressant response, and performs well on key measures of validity (Willner, 1997). Nevertheless, the model has been criticized on both theoretical and practical grounds (Forbes et al., 1996; Barr and Phillips, 1998; Planchez et al., 2019; Markov and Novosadova, 2022), although a recent meta-analysis supported the utility of the model when specifically measuring anhedonia (Antoniuk et al., 2019). Thus, greater confidence should be placed in those studies with mushroom and fungus extracts that measured anhedonia (such as with the SPT) than those that did not. Alternate models of depression were also conducted, such as chronic social defeat stress (Li H. et al., 2021) and maternal separation (Mi et al., 2022), but typically only in a single study; given the importance of reproducibility within this field (Petković and Chaudhury, 2022), the literature will benefit from similar findings from alternate groups, or reproduction by the same groups themselves. Additionally, there are a number of other well-established and commonly used animal models of depression that should be used to assess antidepressant activity with these extracts, including surgical, pharmacological and genetic models (Barr and Phillips, 2002; Song and Leonard, 2005; Barr et al., 2011; Overstreet, 2012; Overstreet and Wegener, 2013; Vollmayr and Gass, 2013; Hendriksen et al., 2015; Czéh et al., 2016; Aleksandrova et al., 2019).

Slightly under half of the studies (22) utilized antidepressant screens such as the FST and TST, rather than models of depression. In most cases, these studies were methodologically sound, and used the appropriate controls, such as concurrent testing for locomotor activity and positive drug controls (Bogdanova et al., 2013; Yankelevitch-Yahav et al., 2015). However, several studies utilized variants of the FST, such as the “weight-loaded” FST (Xu, 2016; Liu Y. et al., 2017), whose validity is less well determined, while one study ascribed antidepressant-like effects based on changes in locomotor activity (Singh et al., 2014), which is a behavior with low specificity for depression. An additional concern was the small proportion of female animals tested, given that major depression is twice as common in women as in men: this issue is prevalent in the field of animal models of neuropsychiatric disorders as a whole (Kokras and Dalla, 2014), but future studies in this area should consider including female animals (Gobinath et al., 2018). Overall, however, the present review suggests that there is significant potential for novel antidepressant drug development with mushroom and fungus extracts provided that models and screens are conducted with high integrity.

## Figures and Tables

**Table:** 

**FST**	forced swim test
**TST**	tail suspension test
**OVX**	ovariectomy
**UCMS**	unpredictable chronic mild stress
**CORT**	corticosterone
**SPT**	sucrose preference test
**CRS**	chronic restraint stress
**CSDS**	chronic social defeat stress
**STZ**	streptozotocin (-induced diabetes)
**PSD**	post-stroke depression
**MS**	maternal separation
**LPS**	lipopolysaccharide
**MCAO**	middle cerebral artery occlusion
**HE**	Hericium erinaceus
**Gl-E**	Ganoderma lucidum extract
**EEGL**	ethanol extract of Ganoderma lucidum
**GLP**	Ganoderma lucidum polysaccharide
**MAK**	Ganoderma lucidum mycelia
**GLT**	Ganoderma lucidum triterpenoid
**AEGI**	aqueous extract of Ganoderma lucidum
**PGL**	Polysaccharide-peptide of Ganoderma lucidum
**GAA**	Ganoderic acid
**PCE**	Psilocybe cubensis extract
**PO**	Pleurotus ostreatus
**EtOH**	ethanol
**MEPS**	exopolysaccharide polysaccharide of Marasmius androsaceus
**PCW**	Poria cocos water extract
**WAM**	water extract of Armillaria mellea
**PSAM**	Protoilludane sesquiterpenoid aromatic esters from Armillaria mellea
**AWE**	Agaricus brasiliensis water extract
**AC**	Antrodia cinnamomea
**Er**	Ergosterol
**CW**	Cordyceps militaris water extract
**COR**	Cordycepin
**PCM**	polysaccharide of Cordyceps militaris
**PTNE**	Paecilomyces tenuipes N45 aqueous extract
**AE**	alcohol extract
**WE**	water extract
**PHC**	Paecilomyces hepiali extract
**OFE**	Ophiocordyceps formosana extract
**BCEF**	bioactive compound from entomogenous fungi
**IR**	intestinal radiation
**E2**	17β-estradiol
**Dp**	depression
**i.p.**	intraperitoneal
**p.o.**	per os (oral)
**i.g.**	intragastric
**s.c.**	subcutaneous
**i.v.**	intravenous
